# Projections of multi-morbidity in the older population in England to 2035: estimates from the Population Ageing and Care Simulation (PACSim) model

**DOI:** 10.1093/ageing/afx201

**Published:** 2018-01-24

**Authors:** Andrew Kingston, Louise Robinson, Heather Booth, Martin Knapp, Carol Jagger, for the MODEM project

**Affiliations:** 1 Institute of Health & Society and Newcastle University Institute for Ageing, Newcastle University; 2 School of Demography, ANU College of Arts and Social Sciences, Australian National University; 3 Personal Social Services Research Unit, London School of Economics and Political Science

**Keywords:** multi-morbidity, future, simulation, ageing, long-term conditions, older people

## Abstract

**Background:**

models projecting future disease burden have focussed on one or two diseases. Little is known on how risk factors of younger cohorts will play out in the future burden of multi-morbidity (two or more concurrent long-term conditions).

**Design:**

a dynamic microsimulation model, the Population Ageing and Care Simulation (PACSim) model, simulates the characteristics (sociodemographic factors, health behaviours, chronic diseases and geriatric conditions) of individuals over the period 2014–2040.

**Population:**

about 303,589 individuals aged 35 years and over (a 1% random sample of the 2014 England population) created from Understanding Society, the English Longitudinal Study of Ageing, and the Cognitive Function and Ageing Study II.

**Main outcome measures:**

the prevalence of, numbers with, and years lived with, chronic diseases, geriatric conditions and multi-morbidity.

**Results:**

between 2015 and 2035, multi-morbidity prevalence is estimated to increase, the proportion with 4+ diseases almost doubling (2015:9.8%; 2035:17.0%) and two-thirds of those with 4+ diseases will have mental ill-health (dementia, depression, cognitive impairment no dementia). Multi-morbidity prevalence in incoming cohorts aged 65–74 years will rise (2015:45.7%; 2035:52.8%). Life expectancy gains (men 3.6 years, women: 2.9 years) will be spent mostly with 4+ diseases (men: 2.4 years, 65.9%; women: 2.5 years, 85.2%), resulting from increased prevalence of rather than longer survival with multi-morbidity.

**Conclusions:**

our findings indicate that over the next 20 years there will be an expansion of morbidity, particularly complex multi-morbidity (4+ diseases). We advocate for a new focus on prevention of, and appropriate and efficient service provision for those with, complex multi-morbidity.

## Introduction

Healthcare delivery was built, and generally remains centred, on the treatment of single diseases. Over the last decade, the growing number of older people (aged 65 years and over) has become a considerable challenge to health and social care service provision and funding, as over 50% have at least two chronic conditions (multi-morbidity) [1, 2]. Moreover numbers of the very old, aged 85 years and over, are set to double over the next 20 years [3], with multi-morbidity the norm in this age group [4]. Multi-morbidity increases the likelihood of hospital admission, length of stay and readmission, raises healthcare costs, reduces quality of life, and increases dependency, polypharmacy and mortality [2, 5]. In addition to multi-morbidity, many of the very old have sensory impairment and incontinence [4], making a single disease-focused model of healthcare unsuitable [6].

Poor health behaviours such as obesity and physical inactivity are risk factors common to a number of diseases, but have received little attention as risk factors for multi-morbidity [5]. Younger cohorts have a higher prevalence of obesity than their equivalents a generation ago [7], which may contribute to the increased prevalence of multi-morbidity in those under 65 years of age [1]. A systematic review suggests that better risk factor management could be the key to improving outcomes for people with multi-morbidity [8]. This is in keeping with NICE guidelines for reshaping treatment for people with multi-morbidity in an all-inclusive framework, with care package delivery accounting for individuals’ disease profiles as a whole [9]. Other risk factors for disease have improved in recent decades, with higher levels of education in more recent cohorts contributing to the reduction in prevalence and incidence of dementia [10].

The contribution of younger cohorts with multi-morbidity as they age into the older population, along with growing numbers of the very old, could dramatically increase the health and social care burden in the coming years [11]. However, reliable projections of the prevalence of, and numbers with, multi-morbidity are lacking since, to date, projection models have focussed either on a single disease (such as coronary heart disease, dementia, or diabetes) [12–14]; on a combination of two diseases (cardiovascular disease and dementia) [15]; or on risk factors (such as obesity and physical activity) rather than disease [16]. The aim of this paper is to examine how key long-term conditions and multi-morbidity will evolve between 2015 and 2035 in the population aged 65 years and over in England, using a new dynamic microsimulation model, Population Ageing and Care Simulation (PACSim).

## Methods

### Model design

We developed a discrete time dynamic microsimulation model PACSim, based on a similar dynamic microsimulation model DYNOPTASim [17] and a previous macrosimulation model [18]. PACSim simulates the survival and characteristics (disease and associated risk factors) of a set of real individuals (the base population) as they age over time, to estimate future prevalence, incidence, and life and health expectancies. Movements between states of each characteristic are determined by applying age, sex and state-specific transition probabilities derived from longitudinal data. Here we provide brief details of the overall design of PACSim and the construction of the base population; further information is available online [19].

### Data sources

Three surveys were combined to create the base population of PACSim: Understanding Society wave 1; the English Longitudinal Study of Ageing (ELSA) wave 5; and the Cognitive Function and Ageing Study (CFAS) II. To enable projections to be made for the population aged 65 years and over up to 2040, the base population includes individuals aged 35 years and over.

### Sociodemographic and health states

Individual characteristics included in PACSim and relevant for this paper fall into three broad categories: sociodemographic (age, sex, marital status, education, socio-economic status); health behaviours (smoking status, physical inactivity, BMI); chronic diseases and geriatric conditions (CHD, stroke, hypertension, diabetes, arthritis, cancer, respiratory disease, dementia, depression, hearing impairment, vision impairment and cognitive impairment). With the exception of dementia, chronic diseases were self-report of doctor-diagnosis. Dementia status was only available in CFAS and was therefore allocated probabilistically, and outwith the simulation, conditional on age group, MMSE category and community/care home residence. Vision and hearing impairments were self-report of current condition and cognitive impairment was defined as a Mini-Mental State Examination (MMSE) score [20] 0–20. Fuller details of data, harmonisation and imputation of missing values are given in online [19].

Individuals’ characteristics are updated monthly over the full time period of the simulation to achieve a more realistic evolution for characteristics which jointly influence each other. All characteristics are stochastic apart from sex, education and socio-economic status which are fixed, and age which is deterministic. Transition models for stochastic characteristics were calculated by fitting binary, ordinal or generalised logistic regression models (dependent upon the characteristic) to the base and 2-year follow-up waves of the combined studies (prior to any imputation for missing values). The coefficients of each model were applied to current characteristics to produce the 2-year probability of moving to a given state; this was converted to a monthly probability. Details of the risk factors included in each transition model are shown in the online report (Table 2) [19]. Dementia did not contribute as an explanatory variable for transition probabilities as dementia status was allocated after the simulation. Monthly survival probabilities were derived from the annual probabilities underlying the 2014-based principal population projection for England [3].

### Model validation

Agreement between the numbers in 5-year age groups at each year of the simulation to the Office for National Statistics 2014 projections for England were good (online report Figure 2) [19]. The age–sex-specific prevalence of stroke, diabetes, current smoking, overweight and obesity from PACSim were compared with those from the Health Survey for England 2014 [21] (online report Table 4) [19]. Generally, there was good agreement apart from the prevalence of obesity where PACSim prevalence was lower by around 8 percentage points for men aged 35–64 and for women of all ages.

### Model outputs

We defined multi-morbidity in two ways: diseases only; and diseases and impairments (vision, hearing, and cognitive impairment no dementia (CIND)—defined as MMSE 0–20 but no dementia), with both measures categorised as none, 1, 2, 3 and 4+ diseases/impairments. Years with and without a disease (or impairment or multi-morbidity) were calculated by year of projection using Sullivan’s method [22], i.e. by applying the age–sex-specific prevalence of the disease/impairment to the age–sex-specific lifetable population generated from the survival probabilities. In addition, using decomposition techniques we derived the relative contributions of rising prevalence of multi-morbidity and increasing life expectancy to changes between 2015 and 2035 in expected years lived with multi-morbidity after age 65 years [23].

Results presented in this paper are predominantly from a single run of PACSim over the time period 2014 to 2035, with the range of values over 10 simulations provided for selected outcomes.

Data harmonisation for the three studies was undertaken in STATA version 12.1, while PACSim was implemented in SAS version 9.4.

## Results

Between 2015 and 2035 increases of more than 50% are projected in the number of older people affected by most individual diseases and impairments, the largest increases being for numbers having cancer (179.4%) and diabetes (118.1%) (Table 1); exceptions are CHD (22.1%), depression (−15.1%) and CIND (25.6%). Arthritis and cancer will see the greatest rise in prevalence of 14.0 and 15.1 percentage points respectively. In the 85+ age group, all diseases apart from dementia and depression more than double in absolute numbers between 2015 and 2035 (see Appendix Table 1 in the Supplementary data, available in *Age and Ageing* online).

**Table 1.** Prevalence of (and numbers with) individual diseases and impairments in 2015, 2025 and 2035 and percentage change in numbers between 2015 and 2025, and 2015 and 2035, population aged 65 years and over

	2015% (*n*)	2025% (*n*)	2035% (*n*)	% Change (2015–2025)	% Change (2015–2035)
Diseases					
Arthritis	48.6 (4,721,300)	60.3 (7,059,300)	62.6 (9,046,300)	49.5	91.6
Cancer	12.6 (1,224,900)	19.6 (2,297,700)	23.7 (3,422,000)	87.6	179.4
CHD	18.3 (1,778,700)	16.6 (1,937,800)	15.0 (2,172,500)	8.9	22.1
Dementia	6.8 (659,700)	7.8 (918,800)	8.5 (1,227,500)	39.3	86.1
Depression	2.3 (225,700)	1.3 (155,500)	1.3 (191,600)	−31.1	−15.1
Diabetes	14.7 (1,428,400)	19.8 (2,317,900)	21.6 (3,115,400)	62.3	118.1
Hypertension	49.0 (4,768,200)	54.9 (6,423,400)	55.9 (8,080,400)	34.7	69.5
Respiratory	18.0 (1,747,400)	21.5 (2,520,000)	24.4 (3,520,300)	44.2	101.5
Stroke	7.5 (726,100)	8.7 (1,021,700)	9.3 (1,337,500)	40.7	84.2
Impairments					
CIND^a^	2.7 (264,100)	2.3 (273,500)	2.3 (331,600)	3.6	25.6
Hearing	12.4 (1,201,800)	11.6 (1,354,400)	12.5 (1,812,400)	12.7	50.8
Vision	6.2 (600,000)	5.2 (613,400)	5.4 (777,700)	2.2	29.6
Total Population	9,723,900	1,170,5800	1,444,9900		

^a^Cognitive impairment no dementia.

### Multi-morbidity

Over half (54.0%) of the population aged 65+ in 2015 have two or more diseases. As expected, multi-morbidity increases with age: in 2015, from 45.7% for those aged 65–74 to 68.7% for those aged 85+; and over time: to 64.4% in 2025 and 67.8% in 2035, for those aged 65+ (Table 2). When four or more diseases are considered, temporal increases at age 65+ occur mostly at age 85+ (Table 2). Although the proportion of the young old (aged 65–74 years) with four or more diseases decreases slightly over time (from 7.0% in 2015 to 6.5% in 2035), the prevalence of multi-morbidity (two or more diseases) is higher in successive young-old cohorts: from 45.7% in 2015 (1941–1950 birth cohort) to 52.8% in 2035 (1961–1970 birth cohort) (Table 2). Similar patterns emerge when multi-morbidity is defined by diseases or impairments (see Appendix Table 2 in the Supplementary data, available in *Age and Ageing* online).

**Table 2.** Prevalence of (and numbers with) multi-morbidity in 2015, 2025 and 2035 and percentage change in numbers between 2015 and 2025 and 2015 and 2035, diseases only, by age group

Age group and number of diseases^a^		2015	2025	2035	% Change	% Change
		%(*n*)	%(*n*)	%(*n*)	(2015–2025)	(2015–2035)
65–74 years	None	20.7 (1,089,600)	16.0 (908,800)	14.5 (1,004,000)	−16.6	−7.9
	One	33.7 (1,777,200)	33.0 (1,875,400)	32.6 (2,253,300)	5.5	26.8
	Two	25.2 (1,329,500)	29.2 (1,657,000)	30.5 (2,107,600)	24.6	58.5
	Three	13.4 (708,500)	14.8 (842,800)	15.8 (1,093,800)	19.0	54.4
	Four or more	7.0 (371,400)	6.9 (392,800)	6.5 (449,000)	5.8	20.9
	Two or more	45.7 (2,409,400)	50.9 (2,892,600)	52.8 (3,650,400)	20.1	51.5
75–84 years	None	11.4 (358,300)	5.9 (252,100)	4.6 (218,200)	−29.6	−39.1
	One	26.7 (835,800)	21.3 (909,100)	19.4 (919,100)	8.8	10.0
	Two	29.8 (932,400)	30.5 (1,299,800)	31.2 (1,473,600)	39.4	58.0
	Three	19.8 (618,500)	24.2 (1,033,600)	26.0 (1,229,600)	67.1	98.8
	Four or more	12.3 (385,000)	18.1 (770,200)	18.8 (886,700)	100.1	130.3
	Two or more	61.9 (1,935,900)	72.8 (3,103,600)	75.9 (3,589,900)	60.3	85.4
85+ years	None	8.8 (115,800)	1.8 (31,700)	0.9 (25,700)	−72.6	−77.8
	One	22.5 (296,700)	10.9 (193,100)	8.5 (240,500)	−34.9	−18.9
	Two	31.5 (415,500)	25.1 (443,500)	21.9 (617,300)	6.7	48.6
	Three	22.3 (293,700)	28.9 (509,000)	28.9 (814,000)	73.3	177.2
	Four or more	14.9 (196,000)	33.3 (586,900)	39.7 (1,117,500)	199.4	470.2
	Two or more	68.7 (905,200)	87.3 (1,539,400)	90.5 (2,548,800)	70.1	181.6
All 65+ years	None	16.1 (1,563,700)	10.2 (1,192,600)	8.6 (1,247,900)	−23.7	−20.2
	One	29.9 (2,909,700)	25.4 (2,977,600)	23.6 (3,412,900)	2.3	17.3
	Two	27.5 (2,677,400)	29.0 (3,400,300)	29.1 (4,198,500)	27.0	56.8
	Three	16.7 (1,620,700)	20.4 (2,385,400)	21.7 (3,137,400)	47.2	93.6
	Four or more	9.8 (952,400)	14.9 (1,749,900)	17.0 (2,453,200)	83.7	157.6
	Two or more	54.0 (5,250,500)	64.4 (7,535,600)	67.8 (9,789,100)	43.5	86.4

^a^Arthritis, cancer, CHD, dementia, depression, diabetes, hypertension, respiratory disease, stroke.

The contribution of mental ill-health (defined as dementia, depression or CIND) to the morbidity burden in 2015 rises strongly with the number of diseases or impairments, with mental ill-health co-existing in 4.1% of those with one other disease or impairment, to 34.1% of those with four or more diseases or impairments (see Appendix Table 3 is available in the Supplementary data, available in *Age and Ageing* online). This pattern is projected to change little between 2015 and 2035 although the contribution of mental ill-health declines slightly over time (see Appendix Table 3 in the Supplementary data, available in *Age and Ageing* online), due to the declines in prevalence of depression and CIND (Table 1).

### Years lived with multi-morbidity

Life expectancy at age 65 for men in 2015 is 18.6 years of which 9.9 years (53.2%) on average will be spent with multi-morbidity (two or more diseases) and 1.9 years (10.0%) with four or more diseases. Women’s life expectancy is longer (21.2 years) with more years spent with two or more diseases (12.2 years, 57.5%) and four or more diseases (2.2 years, 10.6%) (see Appendix Figure 1A in the Supplementary data, available in *Age and Ageing* online). In 2015, most of the remaining years for both sexes will be spent with one or two diseases. Between 2015 and 2035 average years lived free of or with one disease are projected to decrease, and years with multi-morbidity to increase, particularly years with four or more diseases which will double (see Appendix Figure 1A in the Supplementary data, available in *Age and Ageing* online). Similar patterns are seen if impairments are included, though years spent with four or more conditions will form most of the remaining years for both sexes by 2035 (see Appendix Figure 1B in the Supplementary data, available in *Age and Ageing* online).

**Figure 1.**
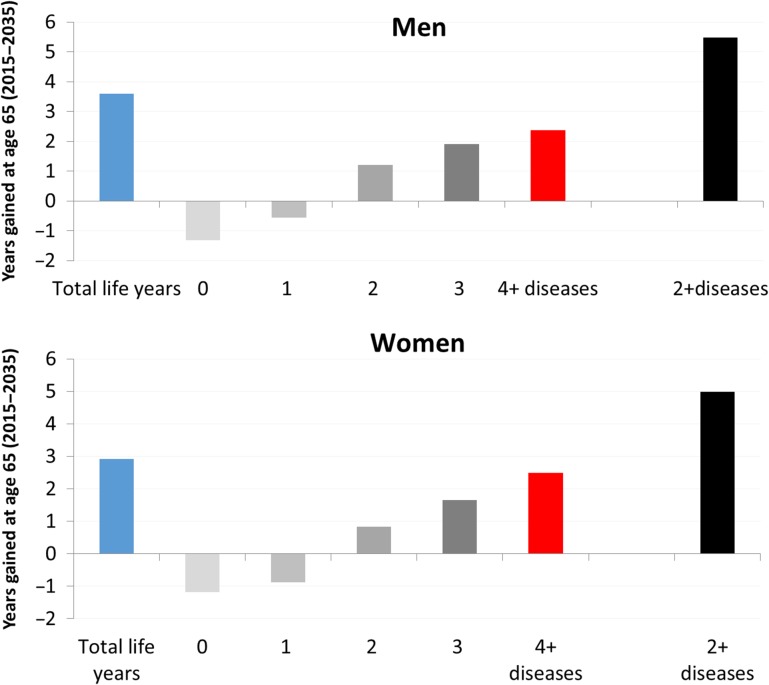
Years gained between 2015 and 2035 in life expectancy at age 65 and years lived from age 65 with multi-morbidity (diseases only), by sex

The gain in life expectancy at age 65 between 2015 and 2035 (men: 3.6 years, women: 2.9 years) comprises a reduction in years spent with no or one disease and an increase in years spent with two or more diseases (men: 5.5 years, women: 5.0 years) and with four or more diseases (men: 2.4 years; women: 2.5 years) (Figure 1). The gain in years lived with two or more diseases will be almost equally a result of longer survival with, and increased prevalence of, multi-morbidity. However, the gain in years lived with four or more diseases will be mainly due to an increased prevalence of multi-morbidity (men: 64.2%, women: 68.4%) (see Appendix Table 4 in the Supplementary data, available in *Age and Ageing* online).

### Sensitivity analyses

In addition to the results presented above derived from a single run of PACSim, a further nine simulations were performed (total *n* = 10). The range in the prevalence of multi-morbidity (defined as two or more diseases, and four or more diseases) were small at less than one percentage point (Appendix Table 5 in the Supplementary data, available in *Age and Ageing* online), as were the ranges in life expectancy and years spent with multi-morbidity at age 65 (Appendix Table 6 in the Supplementary data, available in *Age and Ageing* online).

## Discussion

PACSim provides, for the first time, projections of a range of fatal and non-fatal chronic diseases and geriatric conditions conditional on the sociodemographic characteristics, health behaviours and existing morbidities of a real population aged 35 years and over as they age. We estimate that, over the next 20 years, there will be greater numbers of older people aged 65 years and over, both with individual diseases and with multi-morbidity, particularly with four or more diseases for which numbers will double, as will numbers with cancer, respiratory disease and diabetes. In addition, around a third of those with four or more morbidities will have mental ill-health (depression, dementia or cognitive impairment with no dementia). There will be an expansion of morbidity, with the gain in life expectancy at age 65 between 2015 and 2035 (3.6 years for men, 2.9 years for women) being less than the gain in years spent with multi-morbidity (5.5 years for men, 5.0 years for women), and two-thirds or more of the gain in life expectancy will be spent with four or more diseases. These findings suggest a new focus on those with four or more long-term conditions which we will term ‘complex multi-morbidity’.

## Strengths and limitations

To our knowledge PACSim includes more major chronic diseases than any other microsimulation model, thereby enabling a realistic prediction of the future burden of multi-morbidity. Limitations are focussed around the morbidities included, the time period for calculation of transitions, the assumptions underlying the transitions and the lack of confidence intervals. Firstly, most of the morbidities are self-reported, though all three surveys ascertained doctor-diagnosed disease. The exception to self-reported morbidities are cognitive impairment and dementia, both of which were only available in CFAS and therefore were imputed for participants in the two other surveys. Although PACSim covers the main sociodemographic and lifestyle risk factors for disease, inclusion of other factors was limited by incomparability of items across the three surveys. Secondly, the transition rates for all characteristics were based upon observations from two consecutive waves of each survey, a time period of around 2 years. A longer time period might provide more precise estimates of coefficients in the transition models but we were constrained by CFAS having only two waves and yet being crucial for providing information on dementia and cognitive impairment. Thirdly, the model assumes that transitions between states of all characteristics are independent of time, though incorporating time-varying transitions is a future possibility. Finally, providing confidence intervals around all outcomes that account for the error in the transition rates is non-trivial in a dynamic microsimulation model with as many outcomes and characteristics as PACSim. However running the simulation multiple times has provided evidence that the range of the prevalence of multi-morbidity is small (less than one percentage point) albeit when the error in the transition rates is ignored. Strengths include that: PACSim is based on three large, nationally representative surveys; baseline disease prevalence is broadly comparable with the Health Survey for England 2014 [21]; basing the simulation on monthly transitions which provides more realistic evolutions of characteristics that are co-dependent; and the ability to add scenarios which will allow us to see the effect of future interventions.

PACSim improves upon our earlier macrosimulation model [18] and other models by including real younger individuals with their own health and disease characteristics, rather than assuming that incoming cohorts to the older population have the same characteristics as previous cohorts at 65 years. The higher education levels of real younger cohorts may have a positive effect on some conditions (i.e. lower prevalence) [10], while their higher levels of obesity are likely to have negative consequences [7]. Other projection models assume trends in disease will continue [15]. Given our findings of an increase in multi-morbidity prevalence over time, such assumptions will overestimate the years lived with no or one disease (or impairment) and underestimate the years spent with four or more conditions.

## Comparisons with other studies

Comparisons of the prevalence of multi-morbidity between studies is difficult due to the number and definition of diseases and the age groups included [1, 5]. Nevertheless, our estimate of the prevalence of multi-morbidity of 54% in those aged 65 years and over in 2015 in England is in keeping with others [1]. Risk factors and the prevalence of individual diseases differ between countries, but multi-morbidity is an increasing challenge for all countries, not least low- and middle-income countries (LMIC) in which populations are ageing much more rapidly than in high-income countries [24]. Indeed our finding of a greater likelihood of poor mental health with multi-morbidity, particularly four or more diseases, confirms findings for LMICs [24].

## Policy implications

Geriatricians have long recognised the challenges of multi-morbidity in balancing treatment and intervention options with quality of life and function, particularly in very old and frail people. However, healthcare policy in England has transferred chronic disease management from specialist services to primary care, which through initiatives such as the Quality Outcomes Framework [25] has reinforced a long-standing single-disease paradigm, an approach which does not adequately address the needs of older people. For example, the application of single-disease guidelines from the National Institute for Health and Care Excellence (NICE) for an older person with five conditions (Type 2 diabetes, previous myocardial infarction, osteoarthritis, COPD and depression) may result in a minimum of 11 medications (with up to 10 other drugs routinely recommended), 8–10 routine primary care appointments and 4–6 GP appointments, as well as multiple self-care/lifestyle modifications [26]. Moreover, these findings are not restricted to England, nor to older people: similar levels of polypharmacy and healthcare visits are reported in the US for those in mid-life (aged 45–64 years) with three chronic conditions [27]. The recent NICE guidelines for management of multi-morbidity are, therefore, welcome, especially as they aim to involve patients’ goals and preferences in clinical decision-making [28], though implementation will require training, longer consultations and more funding as primary care, not only in England, is already over-stretched [29].

Our findings indicate that over the next 20 years, the extra years spent with multi-morbidity after age 65 will exceed the gains in life expectancy with an expansion of morbidity, resulting in greater primary and secondary healthcare utilisation [30]. There is also a pressing need to consider the implications for social care, both from formal care services and from unpaid family and other carers, given the strong association between multi-morbidity and reduced functional capacity [30].

We advocate renewed efforts on three fronts: primary prevention by addressing mid- and later-life risk factors; prevention of complex multi-morbidity by targeting older people who have just acquired their second chronic condition; and more efficient future health and social service provision which is more appropriate for people with four or more long-term conditions.

**Key Points**

- Between 2015 and 2035, numbers of older people with 4+ diseases will double and a third of these will have mental ill-health.
- Two-thirds or more of the gain in years of life at age 65 will be years with 4+ long-term conditions (complex multi-morbidity).
- Our findings suggest the need for a focus on prevention of, and service provision for those with, complex multi-morbidity.

## Supplementary Material

Supplementary Data

## Patient involvement

Survey participants were not directly involved in setting the research question or outcome measures or in developing the design or implementation of the study. However, the MODEM Advisory Group, which includes representatives of patient and user organisations and carers, has contributed to the design and has received disseminated results. There are no plans to disseminate results directly to survey participants.
