# Supplementary material for: Projections of multi-morbidity in the older population in England to 2035: estimates from the Population Ageing and Care Simulation (PACSim) model
Source: Age Ageing. 2018 Jan 24;47(3):374–80. doi: 10.1093/ageing/afx201 (PMC5920286; doi:10.1093/ageing/afx201)

**Appendix**

This material formed part of the original submission and has been peer reviewed.

Supplement to: Kingston A, Robinson L, Booth H, Knapp M, Jagger C for the MODEM project. Projections of multi-morbidity in the older population in England to 2035: estimates from the Population Ageing and Care Simulation (PACSim) model. *Age and Ageing* 2018; published online Jan 23. <https://academic.oup.com/ageing/article-lookup/doi/10.1093/ageing/afx201>.

Appendix Table 1: Prevalence of (and numbers with) individual diseases and impairments by age group (65-74, 75-84, 85+) and year (2015, 2025, 2035) from PACSim.

|  |  |  | 2015 %(n) | 2025 %(n) | 2035 %(n) | % Change (2015-2025) | % Change (2015 - 2035) |
| --- | --- | --- | --- | --- | --- | --- | --- |
| **65-74** | Diseases |  |  |  |  |  |  |
|  |  | Arthritis | 43.1 (2273500) | 48.7 (2766200) | 47.5 (3278000) | 21.7 | 44.2 |
|  |  | Cancer | 10.9 (575800) | 14.6 (826300) | 17.0 (1174000) | 43.5 | 103.9 |
|  |  | CHD | 13.9 (735200) | 10.5 (595200) | 9.0 (624900) | -19.0 | -15.0 |
|  |  | Dementia | 2.6 (137100) | 3.8 (214300) | 4.1 (281900) | 56.3 | 105.6 |
|  |  | Depression | 2.9 (151100) | 1.5 (87100) | 1.6 (107800) | -42.4 | -28.7 |
|  |  | Diabetes | 13.7 (720500) | 16.0 (909000) | 16.1 (1114900) | 26.2 | 54.7 |
|  |  | Hypertension | 44.8 (2362700) | 46.9 (2660700) | 46.1 (3184600) | 12.6 | 34.8 |
|  |  | Respiratory | 17.8 (940100) | 18.5 (1052700) | 22.2 (1534800) | 12.0 | 63.3 |
|  |  | Stroke | 5.2 (273500) | 5.0 (282000) | 5.0 (344500) | 3.1 | 26.0 |
|  | Impairments |  |  |  |  |  |  |
|  |  | CIND* | 1.7 (92300) | 2.3 (128000) | 2.3 (159000) | 38.7 | 72.3 |
|  |  | Hearing | 5.5 (289100) | 4.6 (259300) | 4.7 (325200) | -10.3 | 12.5 |
|  |  | Vision | 3.0 (159100) | 2.6 (145800) | 2.6 (181400) | -8.4 | 14 |
| **75-84** | Diseases |  |  |  |  |  |  |
|  |  | Arthritis | 53.3 (1667600) | 67.4 (2875400) | 70.6 (3337700) | 72.4 | 100.1 |
|  |  | Cancer | 14.4 (452200) | 22.8 (971900) | 26.1 (1232100) | 114.9 | 172.5 |
|  |  | CHD | 22.9 (715500) | 19.4 (825600) | 16.1 (761500) | 15.4 | 6.4 |
|  |  | Dementia | 7.7 (240600) | 7.9 (338000) | 8.9 (420400) | 40.5 | 74.7 |
|  |  | Depression | 1.9 (58000) | 1.3 (56300) | 1.3 (61300) | -2.9 | 5.7 |
|  |  | Diabetes | 17.0 (532300) | 22.3 (951700) | 24.2 (1143600) | 78.8 | 114.8 |
|  |  | Hypertension | 54.3 (1698100) | 59.8 (2549500) | 60.8 (2874500) | 50.1 | 69.3 |
|  |  | Respiratory | 19.1 (596700) | 23.5 (1001600) | 24.1 (1139100) | 67.9 | 90.9 |
|  |  | Stroke | 9.0 (282900) | 10.0 (424400) | 9.8 (465400) | 50.0 | 64.5 |
|  | Impairments |  |  |  |  |  |  |
|  |  | CIND* | 3.3 (102100) | 2.4 (100400) | 2.3 (106900) | -1.7 | 4.7 |
|  |  | Hearing | 14.5 (455400) | 11.7 (501000) | 12.0 (566200) | 10 | 24.3 |
|  |  | Vision | 6.5 (204000) | 5.0 (211500) | 5.0 (234100) | 3.7 | 14.8 |
| **85+** | Diseases |  |  |  |  |  |  |
|  |  | Arthritis | 59.2 (780200) | 80.4 (1417700) | 86.3 (2430600) | 81.7 | 211.5 |
|  |  | Cancer | 14.9 (196900) | 28.3 (499500) | 36.1 (1015900) | 153.7 | 415.9 |
|  |  | CHD | 24.9 (328000) | 29.3 (517000) | 27.9 (786100) | 57.6 | 139.7 |
|  |  | Dementia | 21.4 (282000) | 20.8 (366500) | 18.7 (525200) | 30.0 | 86.2 |
|  |  | Depression | 1.3 (16600) | 0.7 (12100) | 0.8 (22500) | -27.1 | 35.5 |
|  |  | Diabetes | 13.3 (175600) | 25.9 (457200) | 30.4 (856900) | 160.4 | 388.0 |
|  |  | Hypertension | 53.7 (707400) | 68.8 (1213200) | 71.8 (2021300) | 71.5 | 185.7 |
|  |  | Respiratory | 16.0 (210600) | 26.4 (465700) | 30.1 (846400) | 121.1 | 301.9 |
|  |  | Stroke | 12.9 (169700) | 17.9 (315300) | 18.7 (527600) | 85.8 | 210.9 |
|  | Impairments |  |  |  |  |  |  |
|  |  | CIND* | 5.3 (69700) | 2.6 (45100) | 2.3 (65700) | -35.3 | -5.7 |
|  |  | Hearing | 34.7 (457300) | 33.7 (594100) | 32.7 (921000) | 29.9 | 101.4 |
|  |  | Vision | 18 (236900) | 14.5 (256100) | 12.9 (362200) | 8.1 | 52.9 |

*CIND = Cognitive impairment no dementia

Appendix Table 2: Prevalence of (and numbers with) multi-morbidity in 2015, 2025 and 2035 and percentage change in numbers between 2015 and 2025 and 2015 and 2035, diseases and impairment, by age group.

|  |  | 2015 | 2025 | 2035 | | % Change | | % Change | |
| --- | --- | --- | --- | --- | --- | --- | --- | --- | --- |
|  |  | %(n) | %(n) | %(n) | | (2015-2025) | | (2015 - 2035) | |
| **Number of diseases and impairments*** | | | | |  | |  | |  |
| 65-74 years | None | 19.5 (1029700) | 15.2 (862200) | 13.8 (955200) | | -16.3 | | -7.2 | |
|  | One | 32.5 (1714700) | 31.8 (1802700) | 31.2 (2153400) | | 5.1 | | 25.6 | |
|  | Two | 24.7 (1304700) | 28.7 (1628500) | 29.9 (2068000) | | 24.8 | | 58.5 | |
|  | Three | 14.1 (746200) | 15.4 (872300) | 16.3 (1127000) | | 16.9 | | 51.0 | |
|  | Four or more | 9.1 (480900) | 9.0 (511100) | 8.7 (604100) | | 6.3 | | 25.6 | |
|  | Two or more | 47.9 (2531800) | 53.1 (3011900) | 54.9 (3799100) | | 19.0 | | 50.1 | |
| 75-84 years | None | 9.7 (303500) | 5.2 (223300) | 4.1 (193300) | | -26.4 | | -36.3 | |
|  | One | 23.7 (742000) | 19.2 (817500) | 17.4 (821200) | | 10.2 | | 10.7 | |
|  | Two | 28.0 (875500) | 28.4 (1212000) | 28.9 (1364100) | | 38.4 | | 55.8 | |
|  | Three | 20.7 (647500) | 24.2 (1033400) | 25.7 (1214300) | | 59.6 | | 87.5 | |
|  | Four or more | 17.9 (561500) | 22.9 (978600) | 24.0 (1134300) | | 74.3 | | 102.0 | |
|  | Two or more | 66.6 (2084500) | 75.5 (3224000) | 78.6 (3712700) | | 54.7 | | 78.1 | |
| 85+ years | None | 5.0 (65700) | 1.2 (20600) | 0.7 (18500) | | -68.6 | | -71.8 | |
|  | One | 16.6 (219200) | 7.5 (131500) | 5.8 (164300) | | -40.0 | | -25.0 | |
|  | Two | 25.2 (331900) | 18.7 (330200) | 16.7 (471200) | | -0.5 | | 42.0 | |
|  | Three | 24.6 (324700) | 25.5 (450600) | 24.6 (692500) | | 38.8 | | 113.3 | |
|  | Four or more | 28.5 (376200) | 47.1 (831300) | 52.2 (1468500) | | 121.0 | | 290.4 | |
|  | Two or more | 78.3 (1032800) | 91.3 (1612100) | 93.5 (2632200) | | 56.1 | | 154.9 | |
| All 65+ years | None | 14.4 (1398900) | 9.4 (1106100) | 8.1 (1167000) | | -20.9 | | -16.6 | |
|  | One | 27.5 (2675900) | 23.5 (2751700) | 21.7 (3138900) | | 2.8 | | 17.3 | |
|  | Two | 25.8 (2512100) | 27.1 (3170700) | 27.0 (3903300) | | 26.2 | | 55.4 | |
|  | Three | 17.7 (1718400) | 20.1 (2356300) | 21.0 (3033800) | | 37.1 | | 76.5 | |
|  | Four or more | 14.6 (1418600) | 19.8 (2321000) | 22.2 (3206900) | | 63.6 | | 126.1 | |
|  | Two or more | 58.8 (5718700) | 67.4 (7893100) | 70.3 (10158600) | | 38.9 | | 79.6 | |

* Arthritis, cancer, CHD, dementia, depression, diabetes, hypertension, respiratory disease, stroke, hearing impairment, vision impairment, cognitive impairment no dementia

Appendix Table 3: Proportion of disease and impairment burden due to mental ill-health defined as dementia, depression or cognitive impairment no dementia (CIND), by year (2015, 2025, 2035), population aged 65 years and over

| Number of diseases and impairments | | Percentage of disease and impairment burden due to mental ill-health | | |
| --- | --- | --- | --- | --- |
|  |  | 2015 | 2025 | 2035 |
|  | One | 4.1 | 2.6 | 2.5 |
|  | Two | 10.0 | 6.5 | 6.4 |
|  | Three | 16.6 | 13.4 | 13.1 |
|  | Four or more | 34.1 | 31.8 | 31.3 |

Appendix Table 4: Decomposed change between 2015 and 2035 in years from age 65 spent with multi-morbidity (two or more disease) and complex multi-morbidity (four or more diseases), by sex

| Life expectancy at age 65 | |  | Men | % | Women | % |
| --- | --- | --- | --- | --- | --- | --- |
|  | With multi-morbidity (2+) | Total | 5.5 | 100.0 | 5.0 | 100.0 |
|  |  | Survival effect | 2.6 | 47.3 | 2.3 | 46.9 |
|  |  | Multi-morbidity effect | 2.9 | 52.7 | 2.6 | 53.1 |
|  |  |  |  |  |  |  |
|  | With multi-morbidity (4+) | Total | 2.4 | 100.0 | 2.5 | 100.0 |
|  |  | Survival effect | 0.8 | 35.8 | 0.8 | 31.6 |
|  |  | Multi-morbidity effect | 1.5 | 64.2 | 1.7 | 68.4 |

Appendix Table 5: Prevalence of multi-morbidity prevalence for 2015, 2025 and 2035 from single simulation and range from 10 simulations, by age group

| Age group and   Number of diseases* | | 2015 | | 2025 | | 2035 | |
| --- | --- | --- | --- | --- | --- | --- | --- |
|  |  | Prevalence (%) | Low – High (%) | Prevalence (%) | Low - High (%) | Prevalence (%) | Low – High  (%) |
| 65-74 years | |  |  |  |  |  |  |
|  | Two or more | 45.7 | 45.4-46.2 | 50.9 | 50.4-50.9 | 52.8 | 52.3-53.3 |
|  | Four or more | 7.0 | 6.8-7.1 | 6.9 | 6.5-6.9 | 6.5 | 6.2-6.6 |
| 75-84 years | |  |  |  |  |  |  |
|  | Two or more | 61.9 | 61.6-62.1 | 72.8 | 72.4-72.8 | 75.9 | 75.7-76.2 |
|  | Four or more | 12.3 | 12.0-12.6 | 18.1 | 17.3-18.2 | 18.8 | 18.4-18.8 |
| 85+ years | |  |  |  |  |  |  |
|  | Two or more | 68.7 | 67.8-68.7 | 87.3 | 86.7-87.3 | 90.5 | 90.3-90.7 |
|  | Four or more | 14.9 | 14.6-15.3 | 33.3 | 32.8-33.5 | 39.7 | 38.8-39.7 |
| All 65+ years | |  |  |  |  |  |  |
|  | Two or more | 54.0 | 53.6-54.3 | 64.4 | 64.0-64.4 | 67.8 | 67.5-67.9 |
|  | Four or more | 9.8 | 9.6-9.9 | 14.9 | 14.5-14.9 | 17.0 | 16.8-17.0 |

* Arthritis, cancer, CHD, dementia, depression, diabetes, hypertension, respiratory disease, stroke

Appendix Table 6: Life expectancy and years spent with two or more and four or more diseases at age 65 for 2015, 2025 and 2035 from single simulation and range from 10 simulations

|  |  | 2015 | | 2025 | | 2035 | |
| --- | --- | --- | --- | --- | --- | --- | --- |
|  |  | Men | Women | Men | Women | Men | Women |
| Age 65 | |  |  |  |  |  |  |
|  | Life expectancy | 18.6 | 21.2 | 20.7 | 22.5 | 22.2 | 24.1 |
|  | Range | 18.6-19.0 | 20.8-21.2 | 20.6-21.0 | 22.5-23.0 | 21.8-22.4 | 23.9-24.5 |
|  |  |  |  |  |  |  |  |
|  | Years with 2+ diseases | 9.9 | 12.2 | 13.5 | 15.3 | 15.4 | 17.2 |
|  | Range | 9.9-10.2 | 11.8-12.2 | 13.3-13.7 | 15.3-15.6 | 14.9-15.4 | 16.9-17.5 |
|  |  |  |  |  |  |  |  |
|  | Years with 4+ diseases | 1.9 | 2.2 | 3.5 | 3.8 | 4.2 | 4.7 |
|  | Range | 1.8-1.9 | 2.1-2.3 | 3.4-3.5 | 3.7-3.8 | 4.1-4.2 | 4.6-4.8 |

Appendix Figure 1: Years lived from age 65 with multi-morbidity based on (A) diseases only, and (B) diseases and impairments, by year (2015, 2025, 2035) and sex

1. Diseases only


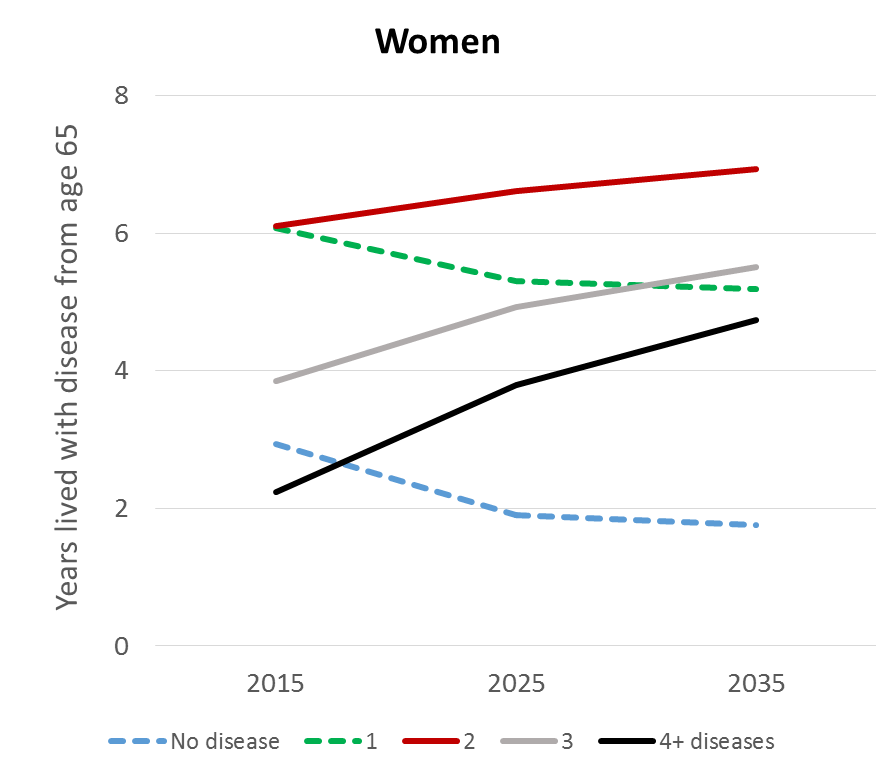

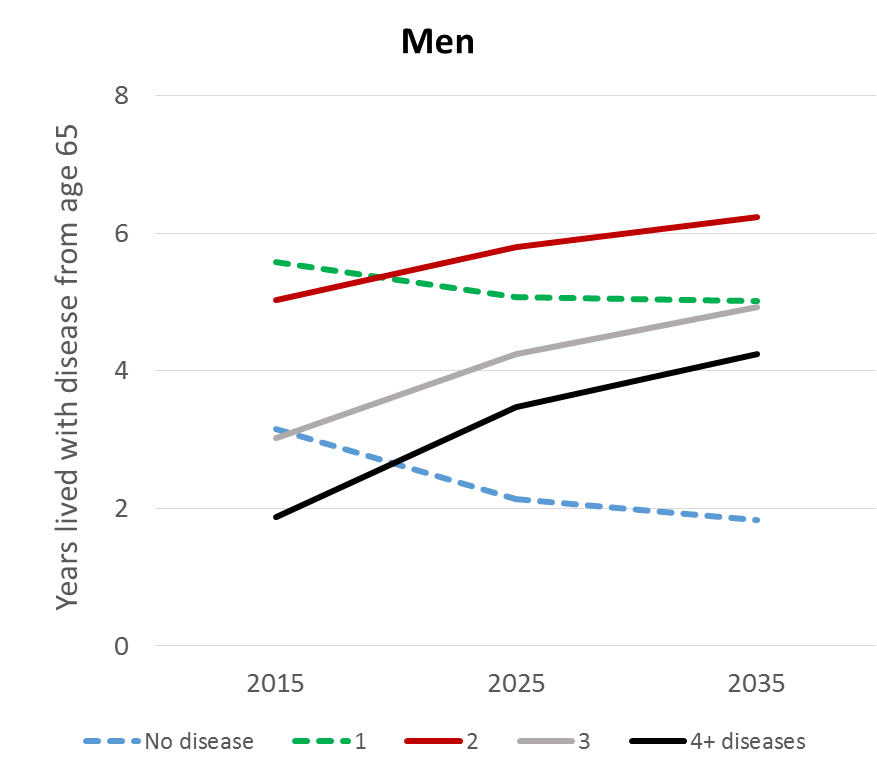


1. Diseases and impairments


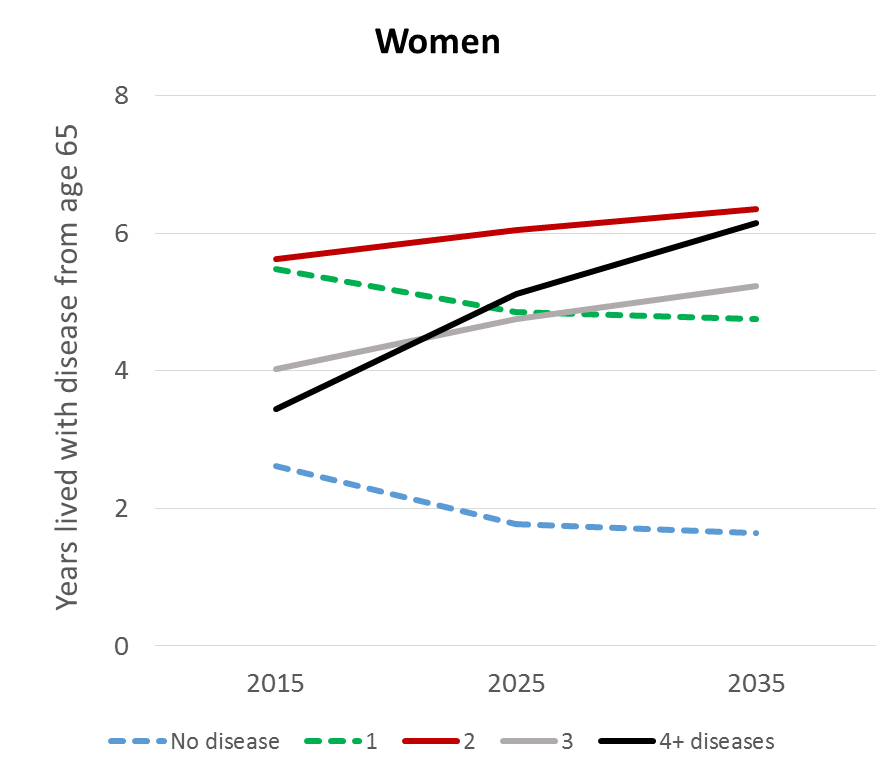

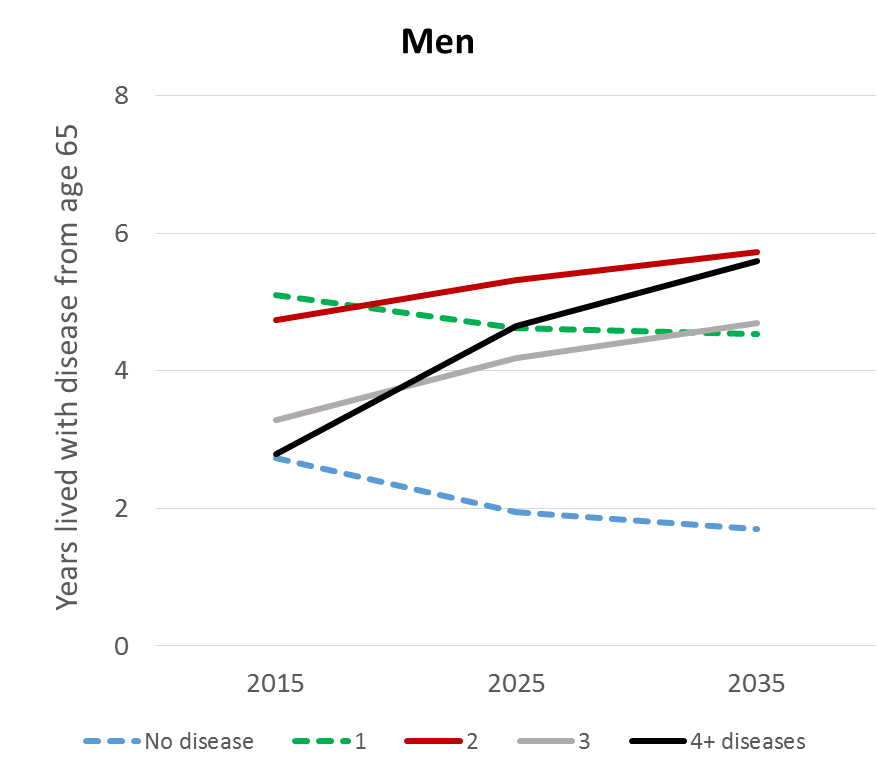

Supplement: Supplementary Data [file appendixmultimorbiditypaper_final2.docx]
